# On the length, weight and GC content of the human genome

**DOI:** 10.1186/s13104-019-4137-z

**Published:** 2019-02-27

**Authors:** Allison Piovesan, Maria Chiara Pelleri, Francesca Antonaros, Pierluigi Strippoli, Maria Caracausi, Lorenza Vitale

**Affiliations:** Department of Experimental, Diagnostic and Specialty Medicine (DIMES), Unit of Histology, Embryology and Applied Biology, University of Bologna, Via Belmeloro 8, 40126 Bologna, BO Italy

**Keywords:** Human genome, Genome length, Genome weight, GC content, Mitochondrial DNA

## Abstract

**Objective:**

Basic parameters commonly used to describe genomes including length, weight and relative guanine-cytosine (GC) content are widely cited in absence of a primary source. By using updated data and original software we determined these values to the best of our knowledge as standard reference for the whole human nuclear genome, for each chromosome and for mitochondrial DNA. We also devised a method to calculate the relative GC content in the whole messenger RNA sequence set and in transcriptomes by multiplying the GC content of each gene by its mean expression level.

**Results:**

The male nuclear diploid genome extends for 6.27 Gigabase pairs (Gbp), is 205.00 cm (cm) long and weighs 6.41 picograms (pg). Female values are 6.37 Gbp, 208.23 cm, 6.51 pg. The individual variability and the implication for the DNA informational density in terms of bits/volume were discussed. The genomic GC content is 40.9%. Following analysis in different transcriptomes and species, we showed that the greatest deviation was observed in the pathological condition analysed (trisomy 21 leukaemic cells) and in *Caenorhabditis elegans*. Our results may represent a solid basis for further investigation on human structural and functional genomics while also providing a framework for other genome comparative analysis.

**Electronic supplementary material:**

The online version of this article (10.1186/s13104-019-4137-z) contains supplementary material, which is available to authorized users.

## Introduction

The genome is the complex of the genetic information of a cell and in eukaryota (and thus in humans) is stored in the nucleus and mitochondria [1]. While mitochondrial DNA (mtDNA) sequence has been known since 1981 [2], the draft sequence of the nuclear human genome was first published in February 2001 [3, 4]. The last human reference genome GRCh38/hg38 was released in December 2013 by the Genome Reference Consortium (GRC) and is the most comprehensive and highest quality mosaic haploid representation compared to previous reference assembly versions, addressing issues about gaps, variants and component and tiling path errors; in addition, for the first time, it contains sequence-based representations for centromeres and telomeres [5, 6].

The fact that very long molecules of human DNA can be contained, following accurate and multiple rounds of folding, within the very limited space of the nucleus, has always attracted attention. In 1990 when sequencing of human genome was just at its beginning, geneticist Jérôme Lejeune affirmed that “*we have got 2 meters of so to speak magnetic tape in which everything is coded*” (Louisiana Legislature, House Committee on the Administration of Criminal Justice, June 7, 1990). Traditionally, it has actually roughly been estimated over the last decades that the total length of human diploid DNA is around 2 m (Table 1) [7–13]. The base composition is usually specified quoting the percentage of guanine (G) and cytosine (C) of a DNA molecule, or GC content [1] and was first estimated through the buoyant density centrifugation [14]. The GC content has been well studied across organisms [15–19], showing its relationships with various genomic characteristics [20–24] and with gene structures such as exons and introns [25–27], for example showing that G-rich repeats are a consistent feature of human ultra-short introns [28, 29].

**Table 1:** Human genome length estimates

References	Meters
Web sites	
http://hypertextbook.com/facts/1998/StevenChen.shtml	Diploid: 1.5–3
http://www.madsci.org/posts/archives/feb99/917376364.Mb.r.html	Haploid: 1
http://book.bionumbers.org/how-big-are-genomes/	Diploid: 2
https://publications.nigms.nih.gov/insidelifescience/genetics-numbers.html	Diploid: 1.8^a^
http://scienceline.ucsb.edu/getkey.php?key=144	Haploid: 1.8
Articles	
[8]	Diploid: 2
[9]	Diploid: 2
[10]	Haploid: 1.5
Books	
[7]	Diploid: 2
[11]	Haploid: 1
[12]	Diploid: 2
[13]	Diploid: 2

^a^This value was converted from the stated 6 feet

The availability of a high-quality reference sequence for the human genome currently offers the possibility to provide an accurate evaluation of these parameters. In this work we propose revised estimations for the length, weight and GC content of the reference human genome and of individual chromosomes, including mtDNA, in a standard human diploid cell and in a reference human being. Moreover, in this paper we discuss the meaning of the obtained results and we formulated a method to calculate the relative GC content in the whole messenger RNA set of sequences and in transcriptomes, comparing different tissues and organisms.

## Main text

### Methods

#### Human genome length and weight calculations

Lengths in centimeters (cm) and weight in picograms (pg) of all 24 human chromosome and mtDNA sequences were calculated as detailed in Additional file 1: Additional Methods.

#### GC content analysis

The genomic GC content was calculated among the certain bases for the 24 chromosomes and for mtDNA as detailed in Additional file 1: Additional Methods.

The “Transcriptomic GC Analysis” (TGCA) software was developed here to study the possible variation of GC content in the expression of whole transcriptomes.

Human quantitative transcriptome maps were previously obtained from publicly available microarray datasets analysed through TRAM (Transcriptome Mapper) software [30] as described [31–33]. Since quantitative gene expression values may anticipate mutational effects that will most likely affect a given human tissue [34], we compared a pathologic cell type with its normal counterpart and a whole organ with one of its subregions (Additional file 1: Additional Methods). For each analysis, only genes for which an expression value is available in both biological conditions were used. For each gene, the longest human mRNA sequence was obtained from the latest version of human 5′_ORF_Extender software [35] (Additional file 1: Additional Methods).

Since TRAM and 5′_ORF_Extender were implemented for other organisms [36, 37], TGCA software itself was implemented with the purpose to be easily used with any sequence and expression dataset of any organism. Thus, we performed GC calculations on other representative species genomes: *Danio rerio*, *Caenorhabditis elegans*, *Saccharomyces cerevisiae* and *Escherichia coli* (Additional file 1: Additional Methods).

### Results

#### Human nuclear genome length and weight

Individual chromosome lengths in bp and cm are given in Table 2. Certain base counts and uncertain base composition estimations given in Additional file 2: Table S1 were used to calculate each chromosome weight, obtaining the results shown in Table 2. The length and weight sums of the 24 chromosomes (22 autosomes and X and Y chromosomes) were used in order to proportionately estimate the length and weight of the unplaced bases, improving whole genome calculation accuracy (Table 2). Data for the previous assembly (GRCh37.p13) are also given in Additional file 3: Table S2 and Additional file 4: Table S3. The chromosomes varying to a greater extent between the two assembly versions are chromosomes 9 and Y (GRCh38 has 2.8 Mb and 2.1 Mb less than GRCh37, respectively) and chromosomes 17 and 18 (GRCh38 has 2.1 Mb and 2.3 Mb more than GRCh37, respectively).

**Table 2:** Length, weight and GC content of human chromosomes, genome and mitochondrial DNA

Chromosome	Length (bp)	Length (cm)	Weight (pg)	Weight (fg)	GC%
1	*248,956,422*	*8.14 *± 0.08	*0.25*	*254.57*	41.72
2	242,193,529	7.92 ± 0.08	*0.25*	247.65	40.23
3	198,295,559	6.48 ± 0.06	0.20	202.76	39.67
4	190,214,555	6.22 ± 0.06	0.19	194.49	*38.24*
5	181,538,259	5.93 ± 0.06	0.19	185.63	39.51
6	170,805,979	5.58 ± 0.05	0.17	174.65	39.61
7	159,345,973	5.21 ± 0.05	0.16	162.94	40.70
8	145,138,636	4.74 ± 0.05	0.15	148.41	40.16
9	138,394,717	4.52 ± 0.04	0.14	141.51	41.28
10	133,797,422	4.37 ± 0.04	0.14	136.81	41.54
11	135,086,622	4.42 ± 0.04	0.14	138.13	41.54
12	133,275,309	4.36 ± 0.04	0.14	136.28	40.77
13	114,364,328	3.74 ± 0.04	0.12	116.94	38.55
14	107,043,718	3.50 ± 0.03	0.11	109.46	40.83
15	101,991,189	3.33 ± 0.03	0.10	104.29	42.03
16	90,338,345	2.95 ± 0.03	0.09	92.38	44.58
17	83,257,441	2.72 ± 0.03	0.09	85.14	45.32
18	80,373,285	2.63 ± 0.03	0.08	82.18	39.78
19	58,617,616	1.92 ± 0.02	0.06	59.95	*47.94*
20	64,444,167	2.11 ± 0.02	0.07	65.90	43.80
21	*46,709,983*	*1.53* ± 0.01	*0.05*	*47.76*	40.94
22	50,818,468	1.66 ± 0.02	*0.05*	51.97	47.00
X	156,040,895	5.10 ± 0.05	0.16	159.55	39.53
Y	57,227,415	1.87 ± 0.02	0.06	58.52	40.03
Total (1–22, X, Y)^a^	3,088,269,832	100.96 ± 0.97	3.16	3157.87	40.87
Unplaced	153,667,028	5.02 ± 0.05	0.16	157.13	
Total male (46, XY)^b^	6,270,605,410	205.00 ± 1.97	6.41	6411.94	40.91
Total female (46, XX)^b^	6,369,418,890	208.23 ± 2.00	6.51	6512.98	40.88
Mean (male and female)	6,320,012,150	206.62 ± 1.99	6.46	6462.46	40.89
mtDNA	16,569	0.00054	0.000017	0.02	44.36
Mean mtDNA per cell	56,793,727	1.86 ± 0.02	0.06	58.08	

Italics: minimum and maximum values

Bp, base pairs; cm, centimeters (variation was calculated considering the uncertainty of the bp number per DNA helical turn [55]); pg, picograms; fg, femtograms; GC%, percentage of G (guanine), C (cytosine) and S (G or C) among certain bases

^a^The total was obtained summing lengths and weights for the 24 types of human linear DNA molecules and used in order to proportionately calculate the length and weight of unplaced bases, improving whole genome calculation accuracy

^b^Total for a male or female diploid cell, including a double complement of unplaced bases

Considering a mean length in a diploid cell of 206.62 cm and the latest estimation of a mean of 3 × 10^12^ nucleated cells for a reference human being [38, 39], the total extension in length of all nuclear DNA molecules present in a single human individual is of about 6.20 billion km (6.20 × 10^12^ m) and is sufficient to cover the Earth-Sun distance (https://cneos.jpl.nasa.gov/glossary/au.html) more than 41 times. Considering a mean weight in a diploid cell of 6.46 pg, the genome weight summed across nucleated human cells would be about 19.39 g, almost the weight of 100 carats (https://sizes.com/units/carat.htm).

#### The mitochondrial genome

Applying all the calculations previously performed for the nuclear genome, the human mtDNA length, weight and GC content were estimated (Table 2).

On average, a human cell could contain from a minimum of 2,845,394 ± 204,296 bp, 0.09 ± 0.0067 cm and 0.0029 ± 0.00021 pg to a maximum of 110,742,060 ± 4,568,736.06 bp, 3.62 ± 0.15 cm and 0.11 ± 0.0047 pg of mtDNA in total, depending on the uncertainty of the number DNA molecules per cell [40] (Additional file 1: Additional Methods). Therefore, the mtDNA, despite its size being greatly reduced in comparison to those of nuclear DNA (1/195,663 compared to haploid nuclear genome), constitutes a significant share of total DNA of a human cell: about 0.90–1.21% (diploid cell), being able to represent at least 52.03% of the DNA in the case of a mature oocyte.

#### GC content analysis

The human GC contents calculated among the certain bases (A, T, W, G, C, and S) counted in the 24 human chromosomes excluding the 150,630,700 uncertain bases are shown in Table 2 (Additional file 4: Table S3 for GRCh37.p13). Among the other investigated species, the calculated chromosome numbers, total genome bp lengths and genomic GC contents (Table 3) are in accordance with previous reports (Additional file 5: Table S4).

**Table 3:** Genomic, mRNA and transcriptomic GC contents in the investigated human conditions and other species

Species	Genomic GC%	mRNA GC%	Transcriptomic GC%	∆GC%^(mRNA−Genomic)^	∆GC%^(Transcriptomic−mRNA)^
*H. sapiens* DS-AMKL	40.89	48.80	48.21	7.91	− 0.59
*H. sapiens* MK			49.27	7.91	0.47
*H. sapiens* Hippocampus		48.83	48.85	7.94	0.03
*H. sapiens* Brain			48.92	7.94	0.09
*D. rerio* Brain	36.63	45.70	45.40	9.08	− 0.30
*C. elegans*	35.44	42.21	43.22	6.77	1.00
*S. cerevisiae*	38.30	39.63	40.21	1.34	0.58
*E. coli*	50.79	51.99	52.13	1.20	0.15

Genomic GC%: percentage of G (guanine), C (cytosine) and S (G or C, present only in *H. sapiens* and *D. rerio* assemblies); for *H. sapiens* the mean GC content between male and female genomes was used; mRNA GC%: percentage of G and C in the analysed messenger RNA set having an expression value together with mRNA sequences available (see “Methods” section for details); transcriptomic GC%: percentage of G and C in the sum of each mRNA GC count multiplied by its mean expression value for each biological condition; ∆GC%^(mRNA−Genomic)^: difference between mRNA and genomic GC%; ∆GC%^(Transcriptomic−mRNA)^: difference between transcriptomic and mRNA GC%

Human Down Syndrome (DS) Acute Megakaryoblastic Leukemia (AMKL) blasts and euploid megakaryoblasts (MK) transcriptome maps have an expression value in both conditions together with mRNA sequences available for 16,547 genes. This value for whole human hippocampus and whole brain transcriptome maps is of 17,579 genes. Among the other investigated species, this value is of 6642 genes for *D. rerio* brain, 19,281 for *C. elegans*, 4673 for *S. cerevisiae* and 2426 for *E. coli*. The mRNA GC contents calculated in these subsets using TGCA software are given in Table 3. For each biological condition, each mRNA GC absolute count was then multiplied by the corresponding expression value. The sum of these values related to each transcriptome map gives the transcriptomic GC content (Table 3). mRNA and transcriptomic GC contents for each chromosome in DS-AMKL and MK conditions are given in Additional file 6: Table S5. DS-AMKL condition has 7 chromosomes (9, 11, 20, 17, 16, 22, 19) with a transcriptomic GC content higher than 48.80 which is the total mRNA GC % (the maximum is 56.26% of chr19), while MK condition has 9 chromosomes (7, 15, 9, 11, 20, 17, 22, 16, 19) with a transcriptomic GC content higher than that value (the maximum is 59.02% of chr19, which is a very high value).

### Discussion

In this work we have determined, to the best of our knowledge, basic parameters describing the normal human reference genome: the length, expressed in terms of both bp and unit of length (cm, m), weight (in unit of mass, pg) and relative GC content expressed in percentages, for the whole human nuclear genome, for each chromosome and for mtDNA.

We have based our calculations on the GRCh38 assembly, which is longer and more contiguous than previous reference assembly versions and provides a sequence-based representation for genomic features such as centromeres and telomeres for the first time [5], which, although variable among cell types and ages, would affect our estimates to a small extent. However, the human genetic diversity ranges from the single-nucleotide variation to large chromosomal events [41, 42]. Following the sequencing of 1000 human genomes [43], a recent analysis estimated ~ 20 million bases of sequence variation in a typical diploid genome [43]. Applying this order of magnitude of variation to our estimates, a proportional variability among individuals of ± 0.65 cm and 0.02 pg for the length and weight of a human mean diploid genome can be assumed.

Our results are not far from previous rough estimates (Table 1), however the more accurate determination of the human genome length and weight might offer interesting possibilities. A recent analysis of 70 genomes from prokaryotes to primates showed that five informational laws about genome structure complexity may have been found [44], suggested by indexes based on the value k = lg_2_(n), where k is the length of a string occurring in the genome and n is the genome length [44]. Applying our analysis to other genomes would be useful to update these indexes. Another interesting possibility offered by the knowledge of human nuclear genome length is the derivation of the total human DNA volume, in order to estimate the efficiency of DNA in data storage, resulted to be in the order of 10^4^ fold superior in comparison to the most currently advanced hard disks (Additional file 7: Discussion). The genome weight is a parameter useful for the correlation with the DNA extraction yields through different methods [45].

Regarding GC content analysis at genomic level, our results are in agreement with a recent study [6]. Through the implementation of TGCA software we have also determined the GC content at mRNA and transcriptomic levels for the first time, a novel concept we propose here, which is the GC percentage calculated in the mRNA amount actually expressed in a tissue. The human genomic GC content results to be much lower than mRNA GC content. mRNA GC content is in turn similar to the transcriptomic GC content. This has been confirmed also in *D. rerio* and *C. elegans* and to a lesser extent in *S. cerevisiae* and in *E. coli*. Overall, it seems that the GC composition of highly and poorly expressed genes in specific tissues affects the mRNA GC content to a small extent and a global compensation between them may exist.

Comparing different biological conditions, the greatest deviation from the mRNA GC content was found in a condition of aneuploidy and leukaemia (DS-AMKL). Interestingly, DS-AMKL transcriptomic GC content skews in a greater extent from the transcriptomic GC content of the healthy euploid counterpart of MK cells. Recent works conducted on DS subjects showed typical alterations of the metabolome and whole transcriptome [46, 47]. Chromosome 21 GC content is one of the closest to the mean genomic GC content, thus the presence of a third copy of chromosome 21 would not cause a great change in GC composition at genomic level. Since the duplication of at least a restricted region of human chromosome 21 is associated to DS [48], further studies are necessary to determine whether the duplication of this chromosome 21 region and/or the leukaemia condition is responsible for this deviation pattern. For example, a recent work showed a high expression of high-GC-content mRNAs in psoriasis lesion transcriptome, while resolving lesions had a low expression of these mRNAs [49]. More in-depth analysis will be needed to validate the use of these indexes as indicators in the comparison of disease versus normal conditions.

Genomic, mRNA and transcriptomic GC content determination can be useful in DNA and RNA sequencing analyses where GC content bias for the Illumina sequencing technology has been documented as likely introduced at the library preparation step, resulting in confounding DNA copy number studies and expression fold-change estimates [50].

In conclusion, we provide an update on fundamental human genome parameters and a first characterisation of the mRNA and transcriptome GC contents. Our results may represent a solid basis for further investigations on human structural and functional genomics [29, 51] while also providing a framework for the comparative analysis of other genomes.

## Limitations

Determination of the length, weight and relative GC content of genome is subjected to the accuracy of the genome assembly and to the variability existing among individuals [41]. Regarding mtDNA, although its sequence has been exactly determined, the mtDNA molecule copy number per cell is of difficult estimation [52]. Regarding GC content at mRNA and transcriptomic levels, the analysis is limited to genes for which an expression value together with the corresponding longest mRNA nucleotide sequence is publicly available. Finally, mRNA 5´ region is known to have a composition bias [53] and a better delimitation of this sequence may be necessary [54].

## Additional files

**Additional file 1: Additional Methods.** Human genome length and weight calculations, human GC content analysis and GC content analysis in other species. Detailed description of the genome length and weight calculations and of the GC content analysis for the human genome and for *Danio rerio*, *Caenorhabditis elegans*, *Saccharomyces cerevisiae*, and *Escherichia coli.*

**Additional file 2: Table S1.** Nucleotide counts in the 24 human chromosomes and estimation of uncertain bases, based on GRCh38.p10. Nucleotide counts for the 24 human chromosomes and estimation of uncertain bases necessary for the genome length and weight calculations and for the GC content analysis, based on the most recent human genome assembly, obtained as described in detail in Additional file 1: Additional Methods file.

**Additional file 3: Table S2.** Nucleotide counts in the 24 human chromosomes and estimation of uncertain bases, based on GRCh37.p13. Nucleotide counts for the 24 human chromosomes and estimation of uncertain bases necessary for the genome length and weight calculations and for the GC content analysis, based on the previous human genome assembly, obtained as described in detail in Additional file 1: Additional Methods file.

**Additional file 4: Table S3.** Length, weight and GC content of human chromosomes, genome and mitochondrial DNA, based on GRCh37.p13. Length, weight and GC content of human chromosomes, genome and mitochondrial DNA, based on the previous human genome assembly, obtained as described in detail in Additional file 1: Additional Methods file.

**Additional file 5: Table S4.** Accordance of our calculations with previous reports. Accordance with previous reports of our calculations of the number of chromosomes and the total genome length for *Danio rerio*, *Caenorhabditis elegans*, *Saccharomyces cerevisiae*, and *Escherichia coli* obtained as described in detail in Additional file 1: Additional Methods file.

**Additional file 6: Table S5.** Genomic, mRNA and transcriptomic GC contents per chromosome in human Down Syndrome (DS) acute megakaryoblastic leukemia (AMKL) blasts and euploid megakaryoblasts (MK) conditions. Genomic, mRNA and transcriptomic GC contents per chromosome in human Down Syndrome (DS) acute megakaryoblastic leukemia (AMKL) blasts and euploid megakaryoblasts (MK) conditions obtained as described in detail in Additional file 1: Additional Methods file.

**Additional file 7: Discussion.** In-depth discussion of obtained results.

## Abbreviations

mtDNA: mitochondrial DNA
IHGSC: International Human Genome Sequencing Consortium
GRC: Genome Reference Consortium
G: guanine
C: cytosine
m: meters
A: adenine
T: thymine
W: A or T
G: guanine
C: cytosine
S: G or C
N: any nucleotide
cm: centimeters
bp: base pairs
nm: nanometers
pg: picograms
kg: kilograms
mRNA: messenger RNA
TGCA: Transcriptomic GC Analysis
TRAM: Transcriptome Mapper
DS: Down Syndrome
AMKL: acute megakaryoblastic leukaemia
MK: megakaryoblasts
Mbp: megabases
Gbp: gigabases
g: grams
CNVs: copy number variants
