# Supplementary material for: On the length, weight and GC content of the human genome
Source: BMC Res Notes. 2019 Feb 27;12:106. doi: 10.1186/s13104-019-4137-z (PMC6391780; doi:10.1186/s13104-019-4137-z)
Supplement: Supplementary file 1 — Additional file 1: Additional Methods. Human genome length and weight calculations, human GC content analysis and GC content analysis in other species. Detailed description of the genome length and weight calculations and of the GC content analysis for the human genome and for Danio rerio, Caenorhabditis elegans, Saccharomyces cerevisiae, and Escherichia coli. [file 13104_2019_4137_MOESM1_ESM.doc]

**On the length, weight and GC content of the human genome**

Allison Piovesan, Maria Chiara Pelleri, Francesca Antonaros, Pierluigi Strippoli, Maria Caracausi* and Lorenza Vitale

**Human genome length and weight calculations**

All 24 human chromosomes (22 automosomes and X and Y chromosomes) and mtDNA sequences were downloaded from the NCBI Nucleotide database using Batch Entrez (https://www.ncbi.nlm.nih.gov/sites/batchentrez) in FASTA format on 23 February 2017 (corresponding to GRCh38.p10, https://www.ncbi.nlm.nih.gov/assembly/GCF_000001405.36/). A Python executable script (http://www.python.org/, version 2.7) which counts all types of nucleotide letters [1] was developed in order to calculate exact base composition of each sequence and is available at http://apollo11.isto.unibo.it/software/. The obtained counts were imported in a suitable basic database, publicly available at http://apollo11.isto.unibo.it/software/, developed within the FileMaker Pro Advanced environment (FileMaker, Santa Clara, CA, version 12) in order to perform the following detailed calculations.

Adenine (A), thymine (T), W (which stands for A or T), guanine (G), cytosine (C) and S (which stands for G or C) are called certain bases here. Other letters including N (which stands for any nucleotide) were counted among the uncertain bases. Certain and uncertain bases were considered for each chromosome and mtDNA calculations. Unlike mtDNA sequence, human nuclear genome assembly also presents unplaced bases, which are sequences not associated with any chromosome (https://www.ncbi.nlm.nih.gov/grc/help/definitions) and thus they have been considered only for calculations performed for the whole genome as explained below.

First, certain and uncertain bases were considered in order to calculate length in centimeters (cm). Unplaced bases have been instead taken into account later. Thus, lengths were calculated for each chromosome separately and for mtDNA using the following basic formula: base pair (bp) count × mean bp interdistance. The mean bp interdistance was calculated assuming 3.4 nanometers (nm) as the length of one DNA helical turn [2] and 10.4 bp ± 0.1 bp per helical turn which was estimated from previous measurements [3].

In order to calculate each chromosome and mtDNA weight in picograms (pg), data related to certain bases were considered while the composition of uncertain bases needed to be proportionately estimated as follows: ATW certain bp : total certain bp = ATW uncertain bp : total uncertain bp. Unplaced bases have been instead taken into account later. The sum of certain ATW and GCS contents and of uncertain calculated base contents were multiplied for the A:T and G:C pair weights of respectively 615.3830 and 616.3711 daltons [4]. These numbers are expressed in dalton (or unified atomic mass unit), which is accepted for use with the International System of Units (http://www.bipm.org/en/publications/si-brochure/table7.html) and is equal to 1.66053892173 × 10^-27^ kilograms or kg (1.66053892173 × 10^-12^ pg).

Finally, the length and weight sums of the 24 chromosomes (22 automosomes and X and Y chromosomes) can at this point be used in order to proportionally estimate the length and weight of the unplaced bases, improving whole genome calculation accuracy.

Male and female nuclear diploid genome length and weight per cell were then calculated considering the sum of lengths and weights of certain, uncertain and unplaced bases.

In order to calculate the total human nuclear genome length and weight, we multiplied the mean between male and female diploid genome lengths and weights for the latest estimation in a reference human being (defined as being between 20-30 years of age, 70 kg in weight and 170 cm in height) of 3 × 10^12^ nucleated cells [5, 6], considering the standard cell as diploid and the number of polyploid and haploid cells negligible.

These calculations were also performed using the same procedure for all 24 human chromosome (22 automosomes and X and Y chromosomes) sequences corresponding to the last patch of the previous assembly (GRCh37.p13).

mtDNA length and weight were multiplied for the latest estimations of the mtDNA molecule copy number per cell which range from a mean of 171.73 ± 12.33 - 2,505.07 ± 328.24 copies to a mean of 772.48 ± 20.96 - 6,683.69 ± 275.74 for energy-intensive tissues [7] and up to at least 100,000 copies in mature human oocytes [8].

All calculations were made considering 15 decimals when available while final results given throughout the text were rounded to 2 decimals. Variation was calculated considering the uncertainty stated in original papers when available.

**Human GC content analysis**

The A, T and W, and the G, C and S counts were used in order to calculate the genomic GC content among the certain bases for the 24 chromosomes and for mtDNA. The genomic GC content was also calculated for the 24 chromosomes of the GRCh37.p13 assembly.

In addition, analogous to the codonome concept which indicates the total number of codons present across all expressed messenger RNAs (mRNAs) in a given biological condition [9], the "Transcriptomic GC Analysis" (TGCA) software was developed here to study the possible variation of GC content in the expression of whole transcriptomes. In one analysis it is possible to compare transcriptomic GC content of two biological conditions.

Human quantitative transcriptome maps were previously obtained from publicly available microarray datasets analysed through TRAM (Transcriptome Mapper) software [10] as described, [11-13] including validation by real-time reverse transcription PCR analysis. TRAM is a software able to integrate and normalise gene expression profile datasets giving a list of linear gene expression values expressed as the mean value of all available values for that locus as a main output [10]. Since quantitative gene expression values may anticipate mutational effects that will most likely affect a given human tissue [14], we have chosen to compare a pathologic cell type with its normal counterpart and a whole organ with one of its subregions. The chosen tissues were Down Syndrome (DS) Acute Megakaryoblastic Leukaemia (AMKL) blasts [12] compared with euploid megakaryoblasts [12] and hippocampus [13] compared with brain [11, 13]. For each analysis, only genes for which an expression value is available in both biological conditions were used. For each gene, the longest human mRNA sequence available was obtained from RefSeq database [15] data previously parsed by the latest version of human 5'_ORF_Extender software [16]. These mRNAs have the most complete sequence information available as annotated on RefSeq records, thus as to the best of our knowledge so far and which is inevitably subject to experimental limitations [17].

First, TGCA software calculates the GC percentage of each mRNA sequence, then giving the total, mean and standard deviation of the mRNA GC content (without accounting for expression values). The absolute GC count of each mRNA is then multiplied by its mean expression value in a given biological condition. The sum of these values is used to calculate the transcriptomic GC content.

TGCA software was developed within the FileMaker Pro Advanced environment (FileMaker, Santa Clara, CA, version 12). Mac OS X and Windows stand-alone software (TGCA versions pre-loaded with transcriptome and sequence data and empty) are freely available to all for basic use at http://apollo11.isto.unibo.it/software/, including the FileMaker runtime and a tutorial. In particular, the tutorial describes how to obtain transcriptome data and mRNA sequences from TRAM and 5'_ORF_Extender software in tabulated text files in detail, respectively, and how to import them in TGCA. Since TRAM and 5'_ORF_Extender were implemented in order to be used for other organisms [9, 18] for example, other analyses can be performed. In addition, TGCA software was implemented with the purpose to be easily used with any sequence and expression dataset of any organism also obtained with other platforms since simple tabulated text files are required to be imported into the software, following the tutorial in detail.

**GC content analysis in other species**

The steps detailed in previous sections were applied in order to perform calculations on other representative species genomes: *Danio rerio* [19], *Caenorhabditis elegans* [20], *Saccharomyces* *cerevisiae* [21, 22] and *Escherichia coli* [23], covering lower-vertebrates as well as invertebrates, unicellular eukaryotes, and prokaryotes.

All types of nucleotide letters were counted in chromosome sequences downloaded from the NCBI Nucleotide database using Batch Entrez (https://www.ncbi.nlm.nih.gov/sites/batchentrez) in FASTA format on 5 July 2017. The A, T and W, and the G, C and S counts were used to calculate the genomic GC content among the certain bases.

In order to investigate possible variations of GC content in the expression of whole transcriptomes of the chosen representative organisms, previously published quantitative transcriptome maps for *D. rerio* brain and for *C. elegans*, *S. cerevisiae* and *E. coli* whole organisms obtained through the TRAM software [10] as described [9] were used and exported as a tabulated text file for each species. The corresponding RefSeq [15] mRNA sequences were previously parsed through the CODONOME software [9] and exported as a tabulated text file for each species. Gene expression and sequence files for each species were then imported into the TGCA software, following the tutorial in detail, in order to obtain the mRNA and the transcriptomic GC contents.

**References**

1. Langtangen HP: A Primer on Scientific Programming with Python, 4 edn: Springer-Verlag Berlin Heidelberg; 2014.

2. Garrett RH, Grisham CM: Biochemistry, 4 edn. Harcourt Brace, Orlando, FL: Saunders College Publishing; 2008.

3. Wang AH, Nathans J, van der Marel G, van Boom JH, Rich A. Molecular structure of a double helical DNA fragment intercalator complex between deoxy CpG and a terpyridine platinum compound. Nature 1978;276:471-474.

4. Dolezel J, Bartos J, Voglmayr H, Greilhuber J. Nuclear DNA content and genome size of trout and human. Cytometry Part A : the journal of the International Society for Analytical Cytology 2003;51:127-128; author reply 129.

5. Bianconi E, Piovesan A, Facchin F, Beraudi A, Casadei R, Frabetti F, Vitale L, Pelleri MC, Tassani S, Piva F *et al*. An estimation of the number of cells in the human body. Ann Hum Biol 2013;40:463-471.

6. Sender R, Fuchs S, Milo R. Revised Estimates for the Number of Human and Bacteria Cells in the Body. PLoS Biol 2016;14:e1002533.

7. D'Erchia AM, Atlante A, Gadaleta G, Pavesi G, Chiara M, De Virgilio C, Manzari C, Mastropasqua F, Prazzoli GM, Picardi E *et al*. Tissue-specific mtDNA abundance from exome data and its correlation with mitochondrial transcription, mass and respiratory activity. Mitochondrion 2015;20:13-21.

8. Shoubridge EA, Wai T. Mitochondrial DNA and the mammalian oocyte. Curr Top Dev Biol 2007;77:87-111.

9. Piovesan A, Vitale L, Pelleri MC, Strippoli P. Universal tight correlation of codon bias and pool of RNA codons (codonome): The genome is optimized to allow any distribution of gene expression values in the transcriptome from bacteria to humans. Genomics 2013;101:282-289.

10. Lenzi L, Facchin F, Piva F, Giulietti M, Pelleri MC, Frabetti F, Vitale L, Casadei R, Canaider S, Bortoluzzi S *et al*. TRAM (Transcriptome Mapper): database-driven creation and analysis of transcriptome maps from multiple sources. BMC Genomics 2011;12:121.

11. Caracausi M, Vitale L, Pelleri MC, Piovesan A, Bruno S, Strippoli P. A quantitative transcriptome reference map of the normal human brain. Neurogenetics 2014;15:267-287.

12. Pelleri MC, Piovesan A, Caracausi M, Berardi AC, Vitale L, Strippoli P. Integrated differential transcriptome maps of Acute Megakaryoblastic Leukemia (AMKL) in children with or without Down Syndrome (DS). BMC Med Genomics 2014;7:63.

13. Caracausi M, Rigon V, Piovesan A, Strippoli P, Vitale L, Pelleri MC. A quantitative transcriptome reference map of the normal human hippocampus. Hippocampus 2016;26:13-26.

14. Caracausi M, Piovesan A, Vitale L, Pelleri MC. Integrated Transcriptome Map Highlights Structural and Functional Aspects of the Normal Human Heart. J Cell Physiol 2017;232:759-770.

15. O'Leary NA, Wright MW, Brister JR, Ciufo S, Haddad D, McVeigh R, Rajput B, Robbertse B, Smith-White B, Ako-Adjei D *et al*. Reference sequence (RefSeq) database at NCBI: current status, taxonomic expansion, and functional annotation. Nucleic Acids Res 2016;44:D733-745.

16. Casadei R, Piovesan A, Vitale L, Facchin F, Pelleri MC, Canaider S, Bianconi E, Frabetti F, Strippoli P. Genome-scale analysis of human mRNA 5' coding sequences based on expressed sequence tag (EST) database. Genomics 2012;100:125-130.

17. Vitale L, Caracausi M, Casadei R, Pelleri MC, Piovesan A. Difficulty in obtaining the complete mRNA coding sequence at 5' region (5' end mRNA artifact): Causes, consequences in biology and medicine and possible solutions for obtaining the actual amino acid sequence of proteins (Review). Int J Mol Med 2017;39:1063-1071.

18. Piovesan A, Caracausi M, Pelleri MC, Vitale L, Martini S, Bassani C, Gurioli A, Casadei R, Solda G, Strippoli P. Improving mRNA 5' coding sequence determination in the mouse genome. Mamm Genome 2014;25:149-159.

19. Howe K, Clark MD, Torroja CF, Torrance J, Berthelot C, Muffato M, Collins JE, Humphray S, McLaren K, Matthews L *et al*. The zebrafish reference genome sequence and its relationship to the human genome. Nature 2013;496:498-503.

20. C. elegans Sequencing Consortium. Genome sequence of the nematode C. elegans: a platform for investigating biology. Science 1998;282:2012-2018.

21. Dujon B. The yeast genome project: what did we learn? Trends Genet 1996;12:263-270.

22. Goffeau A, Barrell BG, Bussey H, Davis RW, Dujon B, Feldmann H, Galibert F, Hoheisel JD, Jacq C, Johnston M *et al*. Life with 6000 genes. Science 1996;274:546, 563-547.

23. Blattner FR, Plunkett G, 3rd, Bloch CA, Perna NT, Burland V, Riley M, Collado-Vides J, Glasner JD, Rode CK, Mayhew GF *et al*. The complete genome sequence of Escherichia coli K-12. Science 1997;277:1453-1462.
