# Supplementary material for: On the length, weight and GC content of the human genome
Source: BMC Res Notes. 2019 Feb 27;12:106. doi: 10.1186/s13104-019-4137-z (PMC6391780; doi:10.1186/s13104-019-4137-z)
Supplement: Supplementary file 4 — Additional file 4: Table S3. Length, weight and GC content of human chromosomes, genome and mitochondrial DNA, based on GRCh37.p13. Length, weight and GC content of human chromosomes, genome and mitochondrial DNA, based on the previous human genome assembly, obtained as described in detail in Additional file 1: Additional Methods file. [file 13104_2019_4137_MOESM4_ESM.doc]

**On the length, weight and GC content of the human genome**

Allison Piovesan, Maria Chiara Pelleri, Francesca Antonaros, Pierluigi Strippoli, Maria Caracausi* and Lorenza Vitale

**Additional file 4: Table S3.** Length, weight and GC content of human chromosomes, genome and mitochondrial DNA, based on GRCh37.p13.

| **Chromosome** | **Length (bp)** | **Length (cm)** | **Weight (pg)** | **Weight (fg)** | **GC%** |
| --- | --- | --- | --- | --- | --- |
| 1 | *249,250,621* | *8.15 ± 0.08* | *0.25* | *254.87* | 41.74 |
| 2 | 243,199,373 | 7.95 ± 0.08 | *0.25* | 248.68 | 40.24 |
| 3 | 198,022,430 | 6.47 ± 0.06 | 0.20 | 202.48 | 39.69 |
| 4 | 191,154,276 | 6.25 ± 0.06 | 0.20 | 195.45 | *38.25* |
| 5 | 180,915,260 | 5.91 ± 0.06 | 0.18 | 184.99 | 39.52 |
| 6 | 171,115,067 | 5.59 ± 0.05 | 0.17 | 174.97 | 39.61 |
| 7 | 159,138,663 | 5.20 ± 0.05 | 0.16 | 162.73 | 40.75 |
| 8 | 146,364,022 | 4.78 ± 0.05 | 0.15 | 149.66 | 40.18 |
| 9 | 141,213,431 | 4.62 ± 0.04 | 0.14 | 144.40 | 41.32 |
| 10 | 135,534,747 | 4.43 ± 0.04 | 0.14 | 138.59 | 41.58 |
| 11 | 135,006,516 | 4.41 ± 0.04 | 0.14 | 138.05 | 41.57 |
| 12 | 133,851,895 | 4.38 ± 0.04 | 0.14 | 136.87 | 40.81 |
| 13 | 115,169,878 | 3.77 ± 0.04 | 0.12 | 117.76 | 38.53 |
| 14 | 107,349,540 | 3.51 ± 0.03 | 0.11 | 109.77 | 40.89 |
| 15 | 102,531,392 | 3.35 ± 0.03 | 0.10 | 104.84 | 42.20 |
| 16 | 90,354,753 | 2.95 ± 0.03 | 0.09 | 92.40 | 44.79 |
| 17 | 81,195,210 | 2.65 ± 0.03 | 0.08 | 83.03 | 45.54 |
| 18 | 78,077,248 | 2.55 ± 0.02 | 0.08 | 79.84 | 39.78 |
| 19 | 59,128,983 | 1.93 ± 0.02 | 0.06 | 60.47 | *48.36* |
| 20 | 63,025,520 | 2.06 ± 0.02 | 0.06 | 64.45 | 44.13 |
| 21 | *48,129,895* | 1.57 ± 0.02 | *0.05* | *49.21* | 40.83 |
| 22 | 51,304,566 | 1.68 ± 0.02 | *0.05* | 52.47 | 47.99 |
| X | 155,270,560 | 5.08 ± 0.05 | 0.16 | 158.77 | 39.50 |
| Y | 59,373,566 | 1.94 ± 0.02 | 0.06 | 60.71 | 39.97 |
| Total (1-22, X, Y)^a^ | 3,095,677,412 | 101.20 ± 0.97 | 3.17 | 3165.45 | 40.90 |
| Unplaced | 139,140,708 | 4.55 ± 0.04 | 0.14 | 142.28 |  |
| Total Male (46, XY)^b^ | 5,976,710,698 | 195.39 ± 1.88 | 6.11 | 6111.43 | 40.95 |
| Total Female (46, XX)^b^ | 6,072,607,692 | 198.53 ± 1.91 | 6.21 | 6209.48 | 40.91 |
| Mean (male and female) | 6,024,659,195 | 196.96 ± 1.90 | 6.16 | 6160.46 | 40.93 |
| mtDNA | 16,569 | 0.00054 | 0.000017 | 0.02 | 44.36 |
| Mean mtDNA per cell | 56,793,727 | 1.86 ± 0.02 | 0.06 | 58.08 |  |

Italics: minimum and maximum values.

Bp: base pairs; cm: centimeters (variation was calculated considering the uncertainty of the bp number per DNA helical turn [55]); pg: picograms; fg: femtograms; GC%: percentage of G (guanine), C (cytosine) and S (G or C) among certain bases. Bold: minimum and maximum values.

^a^ The total was obtained summing lengths and weights for the 24 types of human linear DNA molecules and used in order to proportionately calculate the length and weight of unplaced bases, improving whole genome calculation accuracy.

^b^ Total for a male or female diploid cell, including a double complement of unplaced bases.
