# Supplementary material for: On the length, weight and GC content of the human genome
Source: BMC Res Notes. 2019 Feb 27;12:106. doi: 10.1186/s13104-019-4137-z (PMC6391780; doi:10.1186/s13104-019-4137-z)
Supplement: Supplementary file 5 — Additional file 5: Table S4. Accordance of our calculations with previous reports. Accordance with previous reports of our calculations of the number of chromosomes and the total genome length for Danio rerio, Caenorhabditis elegans, Saccharomyces cerevisiae, and Escherichia coli obtained as described in detail in Additional file 1: Additional Methods file. [file 13104_2019_4137_MOESM5_ESM.doc]

**On the length, weight and GC content of the human genome**

Allison Piovesan, Maria Chiara Pelleri, Francesca Antonaros, Pierluigi Strippoli, Maria Caracausi* and Lorenza Vitale

**Additional file 5: Table S4. Accordance of our calculations with previous reports.**

| **Species** | **Number of chromosomes** | **Total genome length** | **Reference** |
| --- | --- | --- | --- |
| *Danio rerio* | 25 | 1,345,101,833  (of which 4,488,299 uncertain bp) | [1] |
| *Caenorhabditis elegans* | 6 | 100,272,607 | [2] |
| *Saccharomyces cerevisiae* | 16 | 12,071,326 | [3, 4] |
| *Escherichia coli* | 1 | 2,284,124 | [5] |

**References**

1. Howe K, Clark MD, Torroja CF, Torrance J, Berthelot C, Muffato M, Collins JE, Humphray S, McLaren K, Matthews L *et al*. The zebrafish reference genome sequence and its relationship to the human genome. Nature 2013;496:498-503.

2. C. elegans Sequencing Consortium. Genome sequence of the nematode C. elegans: a platform for investigating biology. Science 1998;282:2012-2018.

3. Dujon B. The yeast genome project: what did we learn? Trends Genet 1996;12:263-270.

4. Goffeau A, Barrell BG, Bussey H, Davis RW, Dujon B, Feldmann H, Galibert F, Hoheisel JD, Jacq C, Johnston M *et al*. Life with 6000 genes. Science 1996;274:546, 563-547.

5. Blattner FR, Plunkett G, 3rd, Bloch CA, Perna NT, Burland V, Riley M, Collado-Vides J, Glasner JD, Rode CK, Mayhew GF *et al*. The complete genome sequence of Escherichia coli K-12. Science 1997;277:1453-1462.
