# Supplementary material for: On the length, weight and GC content of the human genome
Source: BMC Res Notes. 2019 Feb 27;12:106. doi: 10.1186/s13104-019-4137-z (PMC6391780; doi:10.1186/s13104-019-4137-z)
Supplement: Supplementary file 7 — Additional file 7: Discussion. In-depth discussion of obtained results. [file 13104_2019_4137_MOESM7_ESM.doc]

**On the length, weight and GC content of the human genome**

Allison Piovesan, Maria Chiara Pelleri, Francesca Antonaros, Pierluigi Strippoli, Maria Caracausi* and Lorenza Vitale

**Discussion**

In this work we have determined, to the best of our knowledge, basic parameters describing the normal human reference genome: the length, expressed in terms of both bp and unit of length (cm, m), weight (in unit of mass, pg) and relative GC content expressed in percentages.

We have found that the male human diploid genome per cell extends for 6.27 Gbp, is 205.00 cm long and weighs 6.41 pg. The female human diploid genome per cell extends for 6.37 Gbp, is 208.23 cm long and weighs 6.51 pg. The estimation for the mean diploid genome of a reference human being, considering the sum of nucleated cells, is 6.20 billion km long and weighs 19.39 g. The genomic GC content is 40.87% (40.91% male, 40.88% female).

Regarding mtDNA, although its sequence has been exactly determined by obtaining the length of 0.00054 cm and the weight of 0.000017 pg per molecule, it is difficult to estimate the mtDNA molecule copy number per cell [1]. However, we have shown that the mitochondrial genome represents a relevant cellular content and this could be useful in studies of several pathologies, including cancer [2] where an especially low number of mtDNA copies has been determined.

We have based our calculations on the GRCh38 assembly, which is longer and more contiguous than previous reference assembly versions and provides a sequence-based representation for genomic features such as centromeres and telomeres for the first time [3], which, although variable among cell types and ages, would affect our estimates to a small extent. However, the human genetic diversity ranges from the single-nucleotide variation to large chromosomal events [4], and it has been recently estimated that in total 217.1 Mbp of the human genome is variable due to copy number variants (CNVs) [5] among phenotypically normal individuals (in contrast to 33.8 Mbp due to single-nucleotide variation). Following the sequencing of 1,000 human genomes [6], recent estimates have evaluated that the difference between a typical individual genome and the reference human genome ranges from 4.1 million to 5.0 million sites. Although almost all of them are single nucleotide polymorphisms and short insertions or deletions, structural variants affect more bases, estimating ~20 million bases of sequence variation in a typical diploid genome [6]. Applying this order of magnitude of variation to our estimates, a proportional variability among individuals of ± 0.65 cm and 0.02 pg for the length and weight of a human mean diploid genome can be assumed.

Our results are not far from previous rough estimates (Table 1), however the more accurate determination of the human genome length might offer interesting possibilities. The length of a genome is expected to be correlated to its biological information content and to organism complexity, although the "c-value paradox" shows that the correlation holds only for lower organisms [7]. Interestingly, a significant relationship between the genome length and virion volume that is not due to geometric constraints nor to the number of proteins has recently been documented in all types of viruses [8]. Genomic complexity among very different organisms cannot be easily described by basic parameters. A recent analysis of 70 genomes from prokaryotes to primates showed that five informational laws about genome structure complexity may be found [9], suggested by indexes based on the value k = lg_2_(n), where k is the length of a string occurring in the genome and n is the genome length [9]. Applying our analysis to other genomes would be useful to update these indexes.

Another interesting possibility offered by the human nuclear genome length knowledge is also the derivation of the human DNA total volume, in order to estimate the efficiency of DNA in data storage. Assuming the nuclear DNA volume as a cylinder containing the double helix of 1.2 nm of radius (23.7 angstrom [10] or 2.37 nm of diameter), the volume for a length of 206.62 cm would be: π × radius^2^ × height (length of genome in µm) = 9.11 µm^3^. Assuming 1 bp = 2 bit, without considering the redundant information contained in the complementary DNA strand, the 6,320,012,150 bp human genome (Table 2) contains 12,640,024,300 bits, which transformed in Gigabytes would be approximately a DVD movie (12.64 Gbits = 1.58 Gbytes, 1 byte being 8 bits). Therefore, in terms of volume/bit, DNA can store 1 bit of information in 0.72 nm^3^. In the currently most advanced hard disks, 1.34 Terabits (i.e. 1,340 Gbits) may be stored in one square inch [11] (i.e. 645.16 mm^2^). Assuming a thickness of 0.01 µm of the film in which data are stored as magnetized particles (conservative estimation), the volume is 6,451,600 µm^3^, turning in 1 bit in 4,814.63 nm^3^, suggesting that efficiency in data storage per volume unit is in the order of 10^4^ fold superior in DNA in comparison to the most currently advanced hard disks.

The genome weight is a parameter useful for the correlation with the DNA extraction yields through different methods. For example, the genome weight has been used by Preis and coll. [12] as standard in order to determine the genomic DNA content extracted from cells and the genomic copy number in polymerase chain reaction experiments allowing the determination of the percentage of transduced cells.

Regarding GC content analysis at genomic level, our results are completely in agreement with a recent study [13]. Through the implementation of TGCA software we have also determined for the first time the GC content at mRNA and transcriptomic levels. We have shown here that the human genomic GC content (40.87%) is much lower than mRNA GC content (48.8%). mRNA GC content is in turn similar to the transcriptomic GC content, a novel concept we propose here, which is the GC percentage calculated in the mRNA amount actually expressed in a tissue. Comparing four different biological conditions: DS-AMKL, MK, hippocampus and brain, we have shown that the greatest deviation from the mRNA GC content is of -0.59% and was found in a condition of aneuploidy and leukaemia (DS-AMKL). Interestingly DS-AMKL transcriptomic GC content (48.21%) skews in a greater extent from the transcriptomic GC content of the healthy euploid counterpart of MK cells (49.27%, with a difference of more than 1%). It should be noted that chromosome 21 GC content is one of the closest to the mean genomic GC content, thus the presence of a third copy of chromosome 21 would not cause a great change in GC composition at genomic level. Since the duplication of at least a restricted region of human chromosome 21 is associated to DS [14], further studies are necessary to determine whether the duplication of this chromosome 21 region and/or the leukaemia condition is responsible for the deviation pattern seen at mRNA versus transcriptomic levels. For example, a recent work showed a high expression of high-GC-content mRNAs in psoriasis lesion transcriptome, while resolving lesions had a low expression of these mRNAs [15]. It would be interesting to investigate if transcriptomic GC content of other pathological conditions shows a similar deviation from the mRNA GC content and from that of the healthy condition.

Applying the GC content analysis for other genomes, deviation of mRNA GC content from genomic GC content has been confirmed also in *D. rerio* and *C. elegans* and to a lesser extent in *S. cerevisiae* and in *E. coli*. Regarding transcriptomic GC contents, *C. elegans* is the species which shows the most variation. In overall, it seems that the GC composition of highly and poorly expressed genes in specific tissues affects the mRNA GC content to a small extent and a global compensation between them may exist.

TGCA software architecture allows the integration with other databases implemented under the same platform useful for future systematic correlation analyses of gene structural and functional data [16, 17]. Furthermore, mRNA 5´ region is known to have a composition bias [18] and a better delimitation of this sequence may be necessary [19]. Finally, genomic, mRNA and transcriptomic GC content determination can be useful in DNA and RNA sequencing analyses where GC content bias for the Illumina sequencing technology has been documented as likely introduced at the library preparation step, resulting in confounding of DNA copy number studies and expression fold-change estimates [20]. Many normalisations in this regard have been proposed and our GC content estimates could be a reference point in these analyses.

**References**

1. Bogenhagen DF. Mitochondrial DNA nucleoid structure. Biochim Biophys Acta 2012;1819:914-920.

2. Reznik E, Miller ML, Senbabaoglu Y, Riaz N, Sarungbam J, Tickoo SK, Al-Ahmadie HA, Lee W, Seshan VE, Hakimi AA *et al*. Mitochondrial DNA copy number variation across human cancers. eLife 2016;5.

3. Schneider VA, Graves-Lindsay T, Howe K, Bouk N, Chen HC, Kitts PA, Murphy TD, Pruitt KD, Thibaud-Nissen F, Albracht D *et al*. Evaluation of GRCh38 and de novo haploid genome assemblies demonstrates the enduring quality of the reference assembly. Genome Res 2017;27:849-864.

4. Alkan C, Coe BP, Eichler EE. Genome structural variation discovery and genotyping. Nature reviews Genetics 2011;12:363-376.

5. Sudmant PH, Rausch T, Gardner EJ, Handsaker RE, Abyzov A, Huddleston J, Zhang Y, Ye K, Jun G, Hsi-Yang Fritz M *et al*. An integrated map of structural variation in 2,504 human genomes. Nature 2015;526:75-81.

6. Auton A, Brooks LD, Durbin RM, Garrison EP, Kang HM, Korbel JO, Marchini JL, McCarthy S, McVean GA, Abecasis GR. A global reference for human genetic variation. Nature 2015;526:68-74.

7. Elliott TA, Gregory TR. What's in a genome? The C-value enigma and the evolution of eukaryotic genome content. Philos Trans R Soc Lond B Biol Sci 2015;370:20140331.

8. Cui J, Schlub TE, Holmes EC. An allometric relationship between the genome length and virion volume of viruses. J Virol 2014;88:6403-6410.

9. Bonnici V, Manca V. Informational laws of genome structures. Sci Rep 2016;6:28840.

10. Dickerson RE, Drew HR, Conner BN, Kopka ML, Pjura PE. Helix geometry and hydration in A-DNA, B-DNA, and Z-DNA. Cold Spring Harb Symp Quant Biol 1983;47 Pt 1:13-24.

11. Rajaraman V, Siva Ram Murthy C: Parallel Computers Architecture And Programming, 2nd edn: Prentice-Hall of India; 2016.

12. Preis M, Schneiderman J, Koren B, Ben-Yosef Y, Levin-Ashkenazi D, Shapiro S, Cohen T, Blich M, Israeli-Amit M, Sarnatzki Y *et al*. Co-expression of fibulin-5 and VEGF165 increases long-term patency of synthetic vascular grafts seeded with autologous endothelial cells. Gene Ther 2016;23:237-246.

13. Guo Y, Dai Y, Yu H, Zhao S, Samuels DC, Shyr Y. Improvements and impacts of GRCh38 human reference on high throughput sequencing data analysis. Genomics 2017;109:83-90.

14. Pelleri MC, Cicchini E, Locatelli C, Vitale L, Caracausi M, Piovesan A, Rocca A, Poletti G, Seri M, Strippoli P *et al*. Systematic reanalysis of partial trisomy 21 cases with or without Down syndrome suggests a small region on 21q22.13 as critical to the phenotype. Hum Mol Genet 2016;25:2525-2538.

15. Swindell WR, Xing X, Voorhees JJ, Elder JT, Johnston A, Gudjonsson JE. Integrative RNA-seq and microarray data analysis reveals GC content and gene length biases in the psoriasis transcriptome. Physiol Genomics 2014;46:533-546.

16. Piovesan A, Caracausi M, Ricci M, Strippoli P, Vitale L, Pelleri MC. Identification of minimal eukaryotic introns through GeneBase, a user-friendly tool for parsing the NCBI Gene databank. DNA Res 2015;22:495-503.

17. Piovesan A, Caracausi M, Antonaros F, Pelleri MC, Vitale L. GeneBase 1.1: a tool to summarize data from NCBI Gene datasets and its application to an update of human gene statistics. Database (Oxford) 2016;2016.

18. Pesole G, Liuni S, Grillo G, Saccone C. Structural and compositional features of untranslated regions of eukaryotic mRNAs. Gene 1997;205:95-102.

19. Vitale L, Caracausi M, Casadei R, Pelleri MC, Piovesan A. Difficulty in obtaining the complete mRNA coding sequence at 5' region (5' end mRNA artifact): Causes, consequences in biology and medicine and possible solutions for obtaining the actual amino acid sequence of proteins (Review). Int J Mol Med 2017;39:1063-1071.

20. Risso D, Schwartz K, Sherlock G, Dudoit S. GC-content normalization for RNA-Seq data. BMC Bioinformatics 2011;12:480.
